# *γ*-Secretase Modulators: Can We Combine Potency with Safety?

**DOI:** 10.1155/2012/295207

**Published:** 2012-12-17

**Authors:** Harrie J. M. Gijsen, Marc Mercken

**Affiliations:** Department of Neuroscience, Janssen Research & Development, Pharmaceutical Companies of Johnson & Johnson, Turnhoutseweg 30, B-2340 Beerse, Belgium

## Abstract

*γ*-Secretase modulation has been proposed as a potential disease modifying anti-Alzheimer's approach. *γ*-Secretase modulators (GSMs) cause a product shift from the longer amyloid-beta (A**β**) peptide isoforms to shorter, more soluble, and less amyloidogenic isoforms, without inhibiting APP or Notch proteolytic processing. As such, modulating *γ*-secretase may avoid some of the adverse effects observed with *γ*-secretase inhibitors. Since the termination of the GSM tarenfurbil in 2008 due to negative phase III trial results, a considerable progress has been made towards more potent and better brain penetrable compounds. However, an analysis of their lipophilic efficiency indices indicates that their increased potency can be largely attributed to their increased lipophilicity. The need for early and chronic dosing with GSMs will require high-safety margins. This will be a challenge to achieve with the current, highly lipophilic GSMs. We will demonstrate that by focusing on the drug-like properties of GSMs, a combination of high *in vitro* potency and reduced lipophilicity can be achieved and does result in better tolerated compounds. The next hurdle will be to translate this knowledge into GSMs which are highly efficacious and safe *in vivo*.

## 1. Introduction

**Figure 1:**
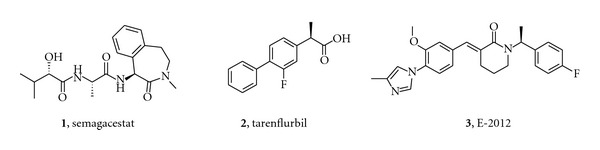


With an ageing population, the prevalence of Alzheimer's disease (AD) and the consequent burden to society are rapidly rising [1]. The currently approved medication for AD only offers symptomatic treatment of limited duration without affecting the progression of the disease. Therefore, disease modifying approaches are urgently needed. A hallmark pathology of AD is the presence of amyloid plaques in the brain, which are mainly aggregates of amyloid-beta (A*β*) peptides of varying length, among which A*β*42 is the most amyloidogenic and neurotoxic. These peptides are formed via sequential, proteolytic processing of the amyloid precursor protein (APP) by two aspartyl proteases: *β*-secretase (BACE) and *γ*-secretase (GS). Consequently, pharmacological intervention in the activity of these secretases has been heavily investigated for over a decade to prevent both the buildup of the amyloid plaques, as well as the formation of toxic amyloid dimers and oligomers [2, 3]. Small molecules inhibiting either BACE or GS offer a direct way to reduce the production of all amyloid peptides. Where the development of *β*-secretase inhibitors has been seriously slowed down/hampered due to the difficulty in achieving adequate brain penetration and concomitant *in vivo* efficacy [4], potent, centrally active *γ*-secretase inhibitors (GSIs) have been discovered and investigated in the clinic and their progress has been reviewed [5, 6]. Phase III clinical trials with the most advanced compound, semagacestat (**1**) (LY450, 139, Figure 1), were prematurely halted in 2010 [6]. Instead of slowing disease progression, **1** was associated with a statistically significant decline in cognition. In addition, an increased risk of skin cancer was reported, most likely related to Notch inhibition.

In comparison with GSIs, *γ*-secretase modulators (GSMs) cause a product shift from the longer amyloid-beta (A*β*) peptide isoforms to shorter, more soluble, and less amyloidogenic isoforms, without inhibiting APP or Notch proteolytic processing. As such, modulating *γ*-secretase may avoid the target-related adverse effects observed upon inhibition of GS.

The identification of a subset of NSAIDs as GSMs in 2001 has led to tarenflurbil **2** (Flurizan) as the first NSAID-derived GSM to be tested in the clinic [7, 8]. After negative results in phase III clinical trials, development was stopped in 2008. This late stage clinical failure can potentially be attributed to the very low brain penetration and weak potency of tarenflurbil [9].

Non-NSAID-derived compounds which do not contain a carboxylic acid group, but are characterized by an imidazole group, were described in a patent application by Neurogenetics in 2004 [10]. Subsequent work by Eisai led to the first compound from this series reaching the clinic, E-2012 (**3**). Although the phase I clinical trial with E-2012 was suspended after lenticular opacity was observed in a high-dose group of a preclinical safety study in rats, in human the compound was reported to reduce plasma A*β*42 levels dose-dependently, with a maximum reduction of ~50% after a 400 mg dose [11].

Since then, many companies have elaborated on these two series, and a considerable progress has been made towards more potent and better brain penetrable compounds in both NSAID- and imidazole-derived chemical series [12, 13]. Despite the efforts spent in this field, very few compounds have made it to the clinic since the termination of tarenflurbil and E-2012. Currently, only two compounds are reported to be in early clinical development: E-2212 and EVP-0962, both with undisclosed structures. In this report, we will provide an overview of our own work and put this into context of the poor drug-like properties of most of the described GSMs to date, especially their high lipophilicity. The need to improve on this in order to obtain safe and efficacious GSMs, suitable for chronic treatment, will also be discussed.

## 2. Methods

### 2.1. Calculation of Efficiency Parameters

Mathematically, ligand efficiency (LE) is calculated according to (1), where Δ*G* represents the binding free energy of a ligand and *n* is the number of heavy (nonhydrogen) atoms. Δ*G* can be calculated using (2), where the dissociation constant *K*_*d*_ can be replaced by IC_50_ [14]. Calculation of LE in this paper was done using a temperature (*T*) of 310 K and using the measured or reported cellular IC_50_ values for the inhibition of A*β*42. As a result, LE is given in kcal/heavy atom according to (3),

(1) $$\begin{array}{c} \text{LE}=\frac{\Delta G}{n}, \end{array}$$

(2) $$\begin{array}{c} \Delta G=-RT\ln K_{d}\approx -RT\ln\left(\text{I}{\text{C}}_{50}\right), \end{array}$$

(3) $$\begin{array}{c} \text{LE}=\frac{1.4\text{pI}{\text{C}}_{50}}{n}. \end{array}$$

Ligand lipophilicity efficiency (LLE) and ligand efficiency-dependent lipophilicity (LELP) were calculated according to (4) and (5), respectively. The octanol-water partition coefficient (clog*P*) is used to describe the lipophilicity of a compound. Log*P* was calculated (clog*P*) using biobyte software.

(4) $$\begin{array}{c} \text{LLE}=\text{pI}{\text{C}}_{50}-\text{clog}P, \end{array}$$

(5) $$\begin{array}{c} \text{LELP}=\frac{\text{clog}P}{\text{LE}}. \end{array}$$

### 2.2. Biology

For the cellular *in vitro* activity of our own compounds, screening was carried out using SKNBE2 human neuroblastoma cells carrying the hAPP 695 wild-type as described in [15]. For a description of the mouse *in vivo* experiments, see references [15, 16]; for details on the dog *in vivo* experiments, see [17].

## 3. Discussion

**Figure 2:**
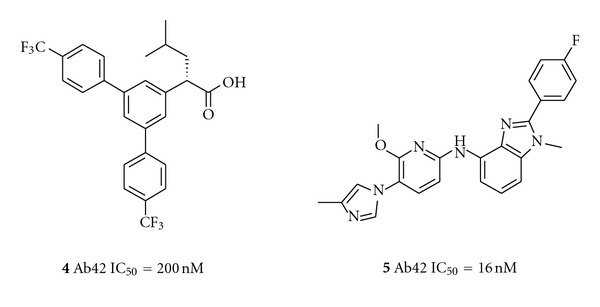


**Figure 3:**
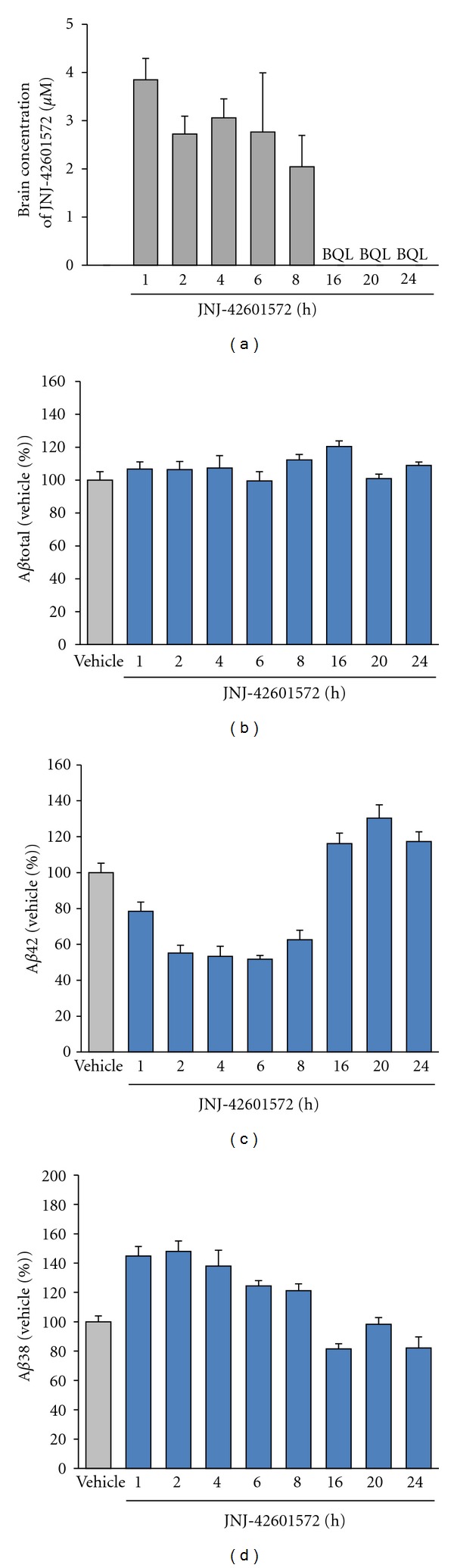
Effects of a single oral dose of 10 mg/kg JNJ-42601572 on A*β* levels in nontransgenic mouse brain as measured with differential ELISAs [16]. Mean (+SEM) brain A*β* levels after drug treatment are expressed as percentage of A*β* levels in brain of vehicle-treated mice. (*n* = 6 mice per data point). BQL: below quantifiable limits (in brain 0.07–0.12 *μ*M).

For both the NSAID-derived carboxylic acid GSMs and the imidazole-derived GSMs, we have reported recently on the activity of optimized, potent submicromolar compounds with good *in vivo* activity in lowering A*β*42 levels in mouse brain and/or dog CSF. The structures of the most profiled compounds are shown in Figure 2. Carboxylic acid JNJ-40418677 (**4**) has lowered A*β*42 in cells with an IC_50_ of 200 nM and had a brain/plasma ratio of 0.5–1.0 in mice, depending on the dose [16]. Imidazole JNJ-42601572 (**5**) lowered A*β*42 in cells with an IC_50_ of 16 nM and had a brain/plasma ratio of 0.7–1.1 in mice [15, 17]. A time profile of JNJ-42601572 in mouse is shown in Figure 3. A similar time profile for JNJ-40418677 has been published [16].

Both compounds have been tested in advanced animal models, including repeated dosing in rat and dog. In these studies, early signs of liver toxicity were noted for both compounds, such as bilirubin and ALT/AST increases [17]. Based on these findings, further development was halted. Since then, the dog model combining PK/PD data with early observations of liver toxicity is routinely used in our discovery program to triage compounds [17]. Both JNJ-40418677 and JNJ-42601572 contain a considerable number of conjugated aromatic rings and are characterized by high lipophilicity and molecular weight. In recent years a number of publications have appeared relating such properties with a low probability of success in clinical development [29, 30]. Catchy phrases like “molecular obesity” and “escape from flatland” have been used to describe the issues associated with high molecular weight and number of (hetero) aromatic rings in molecules [31, 32]. Based on analysis of compound properties and their attrition or success in (pre)clinical development, guidelines have been formulated and proposed which can be applied during optimization efforts in medicinal chemistry programs to strive towards more drug-like compounds. The concept of ligand efficiency (LE) is now routinely applied in the drug discovery process [14]. It is defined as the binding energy towards a biological target per (heavy = nonhydrogen) atom in a compound. Since most assays are not directly measuring binding energy, IC_50_ values are often used to make a relative comparison of the LE of compounds. By working towards a high LE, molecular weight can be kept in control while optimizing pharmacological activity. Additional parameters have been derived, taking the lipophilicity into account: ligand lipophilicity efficiency (LLE) [33] and ligand efficiency-dependent lipophilicity (LELP) [34] are indices which have been proposed to drive the medicinal chemistry towards an acceptable balance between potency and lipophilicity. Striving for optimal values for these parameters in lead optimization should lead to an increase in potency without increasing lipophilicity. For LLE, a value above 5 is desirable, and for LELP, a value below 10 [35]. In this report we will analyze how the GSM field has evolved in regard to lipophilic efficiency and the implications for their developability.

We have calculated the efficiency indices for a number of representative optimized compounds taken from the literature, including our own compounds. A set of acid-derived GSMs are shown in Table 1, and imidazole-containing GSMs are shown in Table 2. Due to the lack of a direct binding assay to GS, we have calculated the ligand efficiency using the pIC_50_ values for reducing A*β*42 in the cellular assay. Cellular activity can be influenced by additional factors, such as permeability, and can therefore differ substantially from the actual binding of the compounds to the GS complex. Nevertheless, by comparing the various efficiency indices within the two respective chemical classes, we feel the calculated values can still be used to rank order compounds.

In Table 1, the acid-derived GSMs are shown in more or less chronological order. Starting with tarenflurbil, the slightly more potent and brain penetrant CHF5074 is followed by more recently published and considerably more potent analogues JNJ-40418677, BIIB042, and compounds **8** and **9** from GSK and Merck, respectively. The optimization efforts around tarenflurbil have led to an increase in potency and brain penetration, but the ligand and lipophilic efficiency indices for these compounds have hardly been improved. LLE has remained very low for **5**, **6**, and **8**, and although an improvement is seen for **7** and **9**, the values are still below the desirable level (>5). For LELP, all compounds have values well above the desirable upper limit of 10, and no compounds show improvement over tarenflurbil. The increase in potency of GSMs **5**, **7**, and **8** can mainly be attributed to an increase in lipophilicity. For **9**, the lipophilicity has not increased compared to tarenflurbil, and **9** also has a reduced aromatic ring count, compared to the other analogs. However, potency and brain penetration for this compound remain suboptimal. Regarding these metrics, tarenflurbil is still the most drug-like molecule, and indeed the compound was well tolerated during clinical trials despite high dosage [36]. In addition to tarenflurbil, of this set of compounds only the closest analogue, CHF5074 (**6**), has progressed into clinical trials. For all other compounds in Table 1 no further development has been reported.

A similar picture emerges for the nonacidic GSMs, mostly containing an imidazole moiety (Table 2). The early neurogenetic compound **10** has a relatively high ligand efficiency of 0.37 but is characterized by very high lipophilicity, resulting in a low LLE and high LELP value. Going from **10** to E-2012 (**3**), both LLE and LELP have improved. Subsequent work around this series by various companies, as exemplified in Table 2 by 11–15 and **5**, has not resulted into a significant further improvement of values relevant for drug-likeness, and in most cases a deterioration of them. The medicinal chemistry program towards **15** was aimed at optimizing lipophilic efficiency [28], which has resulted in the best values for clog*P*, LLE, and LELP within the set of compounds shown in Table 2. However, potency of this compound is still moderate, resulting in a 36% lowering of A*β*42 levels in Guinea pig brain after a 100 mg/kg oral dose [28]. From the compounds in Table 2, only compound **3** (E-2012) has progressed into the clinic. After a single dose of 80 mg/kg of **3** in dog, we have observed liver parameter changes as well as large changes in gene-expression profile in liver tissue obtained in this study [17]. The still suboptimal values for lipophilic efficiency of **3** may have contributed to this, as well as to the termination of **3** in phase 1 clinical trials.

From the *in vivo* data obtained with JNJ-40418677 [16], as well as for JNJ-42601572 shown in Figure 3, two relevant observations can be made, which are also apparent from all other GSMs we have tested *in vivo* up to now. The first observation is that reduction of brain A*β*42 nicely correlates with the compound levels in brain. After the disappearance of the compound from the brain, the A*β* levels quickly return to baseline. This implies that compound levels need to be sustained to maintain a desired change in A*β* levels. The second observation is the large difference between the *in vitro* potency and the compound concentrations required to reduce A*β*42 levels *in vivo*. A considerably higher total compound concentration is required than the *in vitro* IC_50_ to achieve a significant reduction of A*β*42 levels *in vivo*. A common practice is to correct the plasma and brain concentration for the fraction bound to plasma proteins or brain tissue and to use the free compound concentrations. Both JNJ-40418677 and JNJ-42601572 are highly protein/brain tissue bound, with free fractions in plasma and brain of less than 0.1%, resulting in free compound concentrations below the *in vitro* (cellular) IC_50_ values. However, it should be noted that free fractions below 1% are often difficult to be determined accurately, with minor changes in absolute values leading to major differences in calculated free concentrations. After dosing of JNJ-42601572 in dog, the compound concentration in CSF has also been measured, which can be considered as a surrogate for free brain concentrations. After an oral dose of 20 mg/kg, compound levels were only measurable at the plasma Cmax of 4 h, with a CSF/plasma ratio of 0.003. This corresponds to a CSF compound concentration of 11 ± 5 nM, in the same range as the *in vitro* cellular potency (IC_50_, 16 nM). An increase in free brain concentration as measured by the fraction unbound in brain (*F*_ub_) could potentially lead to an improved *in vivo* reduction in A*β*42. A good correlation exists between *F*_ub_ and the lipophilicity of a compound (Log*P*) [37], giving another reason to aim for a reduced lipophilicity of the compounds. The high lipophilicity required to achieve potency, as evident from all the published GSMs and data shown in Tables 1 and 2, is likely related to the membrane embedded character of the GS proteins and the site where these modulators interact with these proteins. This poses the question if the nature of the target allows for potent compounds with a low lipophilicity.

Since JNJ-42601572, our GSM program has focused more strongly on improving the drug-like properties, while maintaining good *in vivo* activity. In the design of the next generation of GSMs, we targeted the following profile of the compounds:

- lower lipophilicity,
- lower molecular weight,
- reduced aromaticity,
- higher solubility,
- higher free fraction.

**Figure 4:**
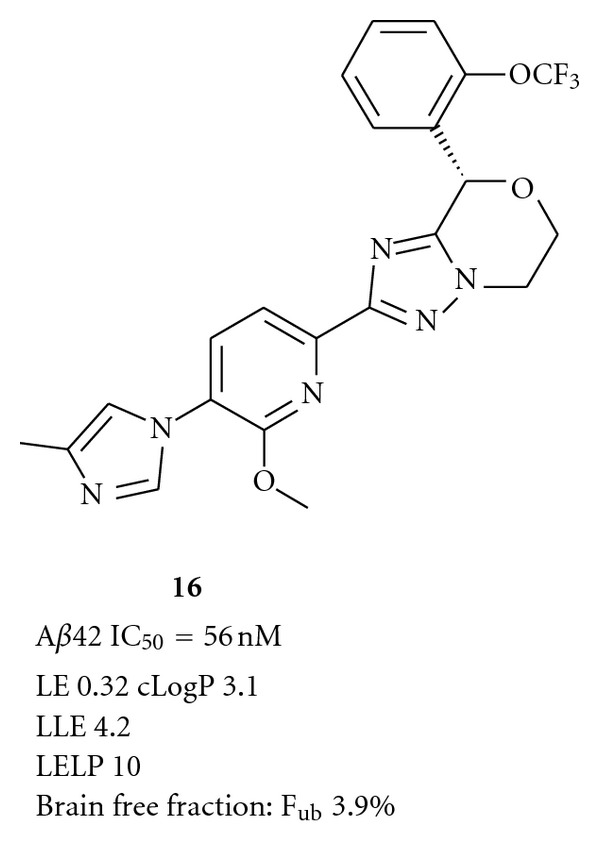
“Next generation” GSM.

A representative structure arising from these efforts is shown in Figure 4, including key *in vitro* data and relevant calculated parameters. (The detailed lead optimization toward compound **16** will be published elsewhere in due course.) 

Although the ligand efficiency of this compound has not improved compared to the previously described structures, the reduced lipophilicity results in a pronounced improvement of LLE and LELP. Also the brain free fraction has increased considerably compared to previous lead JNJ-42601572. When dosed in our dog model at 20 mg/kg p.o., this compound displayed a clear reduction in CSF A*β*42 of 30%–40% at 4 and 8 h after dosing. Despite the high exposure levels in plasma (Cmax at 4 h of 24 ± 3 *μ*M), no increases in bilirubin or ALT levels were observed. In a subsequent limited tolerance study, a two-week daily dose of 200 mg/kg in rats did not lead to overt signs of liver toxicity. These results strengthened our belief that liver toxicity findings with previous GSMs were not target related and that it should be feasible to develop GSMs without liver toxicity.

For **16**, despite the improved free fraction in brain, still high concentrations were required to achieve a significant lowering of A*β*42. CSF compound levels at 4 h were 524 ± 62 nM, considerably above the cellular IC_50_ of 56 nM. This discrepancy is still not fully understood but has also been observed with other analogues with reduced lipophilicity and increased *F*_ub_.

Although a lowering of lipophilicity may therefore not necessarily lead to an increased *in vivo* activity at lower total compound concentrations, the improved profile on liver toxicity parameters of the less lipophilic GSMs has warranted further optimization in this direction.

**Figure 5:**
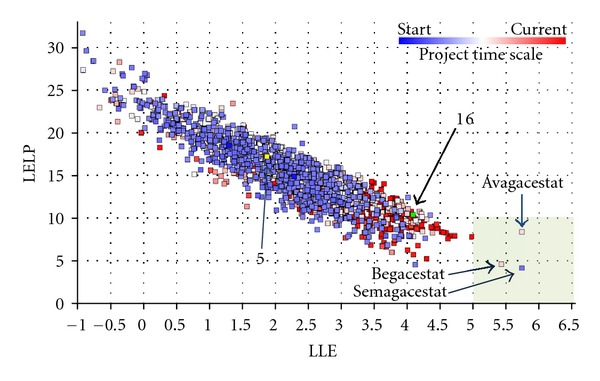
Evolution of our non-acid GSM series in relation to lipophilic efficiency parameters LELP and LLE. (a) LELP and LLE were calculated as described in Section 2. The highlighted section (LELP < 10, LLE > 5) indicates the desired area for drug-like compounds [35]. Each square indicates a compound, with the colour being indicative for when the compound has been synthesized during the project.

In Figure 5 the evolution in our nonacidic GSM program is plotted in regard to the lipophilic efficiency parameters LLE and LELP. The square in the right bottom corner indicates the desired area with optimal LLE and LELP for successful development related to compound safety and quality, in analogy with the analysis of Tarcsay et al. [35]. The individual squares represent the non-acid GSM compounds prepared and tested in our program with a cellular A*β*42 IC_50_ below 5 *μ*M. The colour is representative for when they were synthesized during the project. Chronologically, going from blue-white-pink to red, the red compounds have been prepared most recently. The yellow dot indicates JNJ-42601572 (**5**), while the green dot indicates **16**. Considering the increased presence of recently prepared (red) compounds closer to the optimal region of the plot, clearly progress has been made towards higher-quality compounds. Within the depicted compounds, only three compounds lie within the desired area. These have been added as reference and are actually three of the GSIs which have been or are still in clinical development: semagacestat, begacestat, and avagacestat. Indeed, the toxic side effects observed with these compounds in the clinic are not related to compound quality but more to target-related (mechanism-based) side effects.

The progress in lipophilic efficiency demonstrates that it is possible to make GSMs with reduced lipophilicity while retaining a good cellular potency. The requirement of high compound exposure to achieve *in vivo* efficacy to change A*β* levels in brain and CSF remains a challenge. CNS targeting compounds, and certainly the more lipophilic ones, tend to have high tissue distribution towards other fatty tissue such as liver, increasing the chance for liver damage further. For example, in a tissue distribution study of JNJ-42601572 in rats, a liver/plasma ratio of 8.5 was observed. According to the prevalent theory, an early and likely chronic intervention will be required in order to be effective against AD, certainly for an amyloid cascade targeting a compound like GSM [38]. The safety demands for such a drug will be considerable. A chronic treatment at high, micromolar compound levels, as currently required for significant changes in A*β* levels, is likely to result in adverse effects in at least part of the patient population. How much change in A*β* levels is required to be therapeutically beneficial is still an open question and will certainly influence safety margins for any GSM compound. Chronic treatment of transgenic mice with JNJ-40418677 had a preventive effect on plaque load at compound exposure levels below concentrations required for a reduction in A*β*42 after a single dose [16]. A lower dose than required for acute reduction of A*β*42 levels will translate into increased safety margins. On the other hand, a preventive approach for AD using a GSM potentially requiring lifelong, chronic treatment will certainly increase the safety demand further.

Clearly, a next generation of GSMs beyond the structures discussed in this paper will be needed. Ideally, they should be efficacious *in vivo* at strongly reduced compound concentrations or at least demonstrate significant safety margins. Our efforts in this field have shown progress in optimizing the drug-like properties of GSMs, especially in reducing lipophilicity. Nevertheless, it is clear that we are working at the boundaries of druggable space, with the physicochemical properties required for a highly efficacious GSM conflicting with those required for drug-likeness. A next breakthrough finding may be required to deliver the quality compounds required to test the GSM approach in humans and, if successful, to make it to the market. This will require perseverance in the study of GS and its modulation. Perhaps the evolution of the BACE inhibitor field can serve as an example here: while potent and selective BACE inhibitors were prepared just a few years after the discovery of the BACE enzyme, a decade-long struggle followed to identify brain penetrant, *in vivo* efficacious compounds. But this has ultimately been paid off, and key structural requirements have been identified to achieve good brain penetration [39]. Now, an increasing number of BACE inhibitors are moving forward in(to) the clinic. Unfortunately, in contrast to BACE, no X-ray structures are available of the membrane embedded *γ*-secretase protein complex to allow for structure-based design. More insight into the precise molecular mechanism of GSMs could be of great help in further optimization. Several photoaffinity labelling studies with carboxylic acid [40, 41] and imidazole [42] derived GSMs have now been reported, suggesting that compounds of both classes bind to the N-terminal fragment of presenilin. This does not preclude additional interactions with membrane lipids or the membrane embedded amino acid residues of APP, and hopefully future research will pinpoint the site of action more precisely.

Novel chemical series of GSMs have started to appear, such as natural product-derived triterpene derivatives [43, 44]. Although these are again highly lipophilic and high molecular weight compounds, aromaticity is strongly reduced. Their different profile in A*β*-level modulation may be the result of an alternative interaction or a binding site within GS and illustrate that there may be a potential for additional chemical space to modulate the activity of GS.

## 4. Concluding Remarks

In regard to the intervention in amyloid peptide production, *γ*-secretase modulation can be clearly distinguished from the straightforward inhibition by either GS or BACE. GSMs have actually been proposed as GS activators by enhancing the cleavage of the longer, more amyloidogenic peptides towards shorter amyloid peptides A*β*39-A*β*37 [45]. As such, GSMs may counter the loss of GS function linked to many familial AD causing mutations [46]. Another clear differentiation of GSMs compared to BACE and GS inhibitors lies in the multiple proteins processed by BACE and GS, and the potential adverse effects when inhibiting the processing of these proteins. Where inhibition of BACE has so far not been related to overt toxicities, inhibition of GS has led to especially Notch-related toxicity. Based on these distinctions, GS modulation deserves an optimal effort to obtain drugable, high quality GSMs. Where the principle of modulation makes on-target side effects less likely, the potential for off-target-related side effects is considerable with the currently required high, micromolar concentrations of relatively lipophilic compounds. With the compounds from the chemical series described in this paper, the question in the title “can we combine potency with safety?” cannot be answered positively yet. Nevertheless, progress towards less lipophilic, more drug-like GSMs has been made. A next generation of increased drug-likeness, potency, and/or *in vivo* efficacy, will be required. Further insight into the structure of the GS complex and the interaction with GSMs may provide the required insight on how to get to novel chemical space. In addition, the use of ligand efficiency parameters as discussed in this paper and other drug-likeness guidelines during the optimization process will be crucial to ultimately deliver a high quality-GSM.

## Figures and Tables

**Table 1:** Key parameters and efficiency indices of a set of representative acid GSMs.^a^

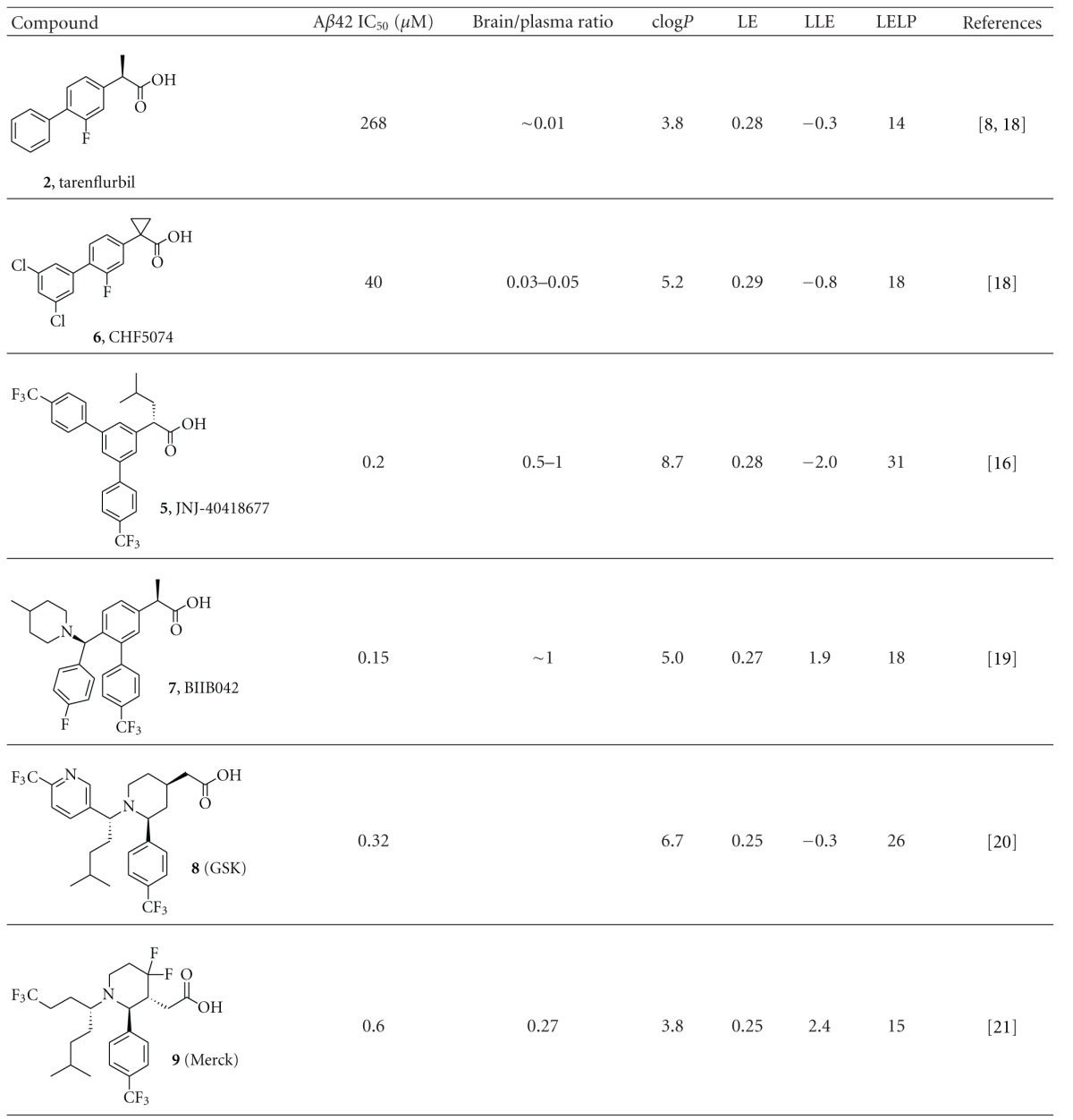

^a^LE, LLE, and LELP were calculated using the formulas given in the Methods section. *In vitro* potency values were taken from the indicated references.

**Table 2:** Key parameters and efficiency indices of a set of representative nonacid GSMs.^a^

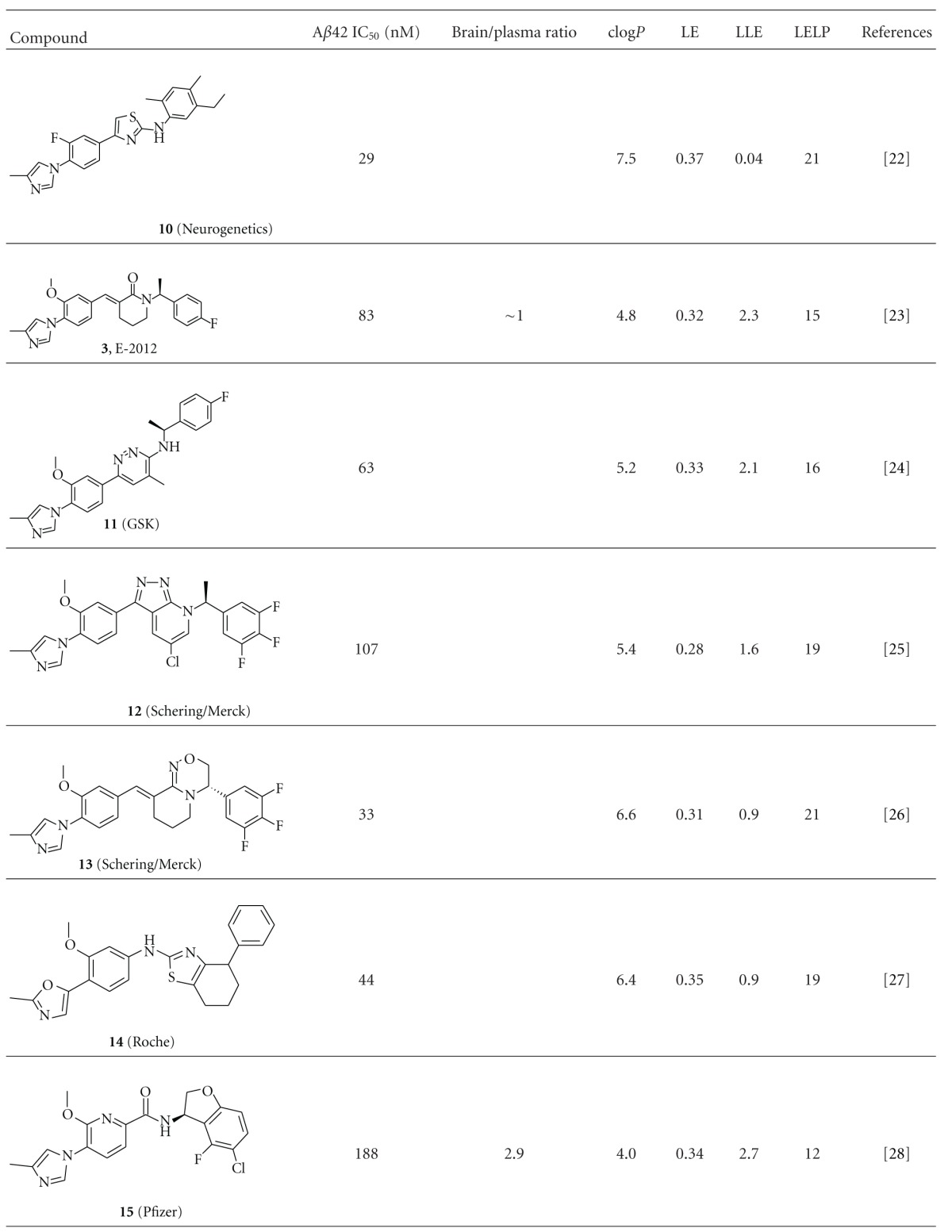 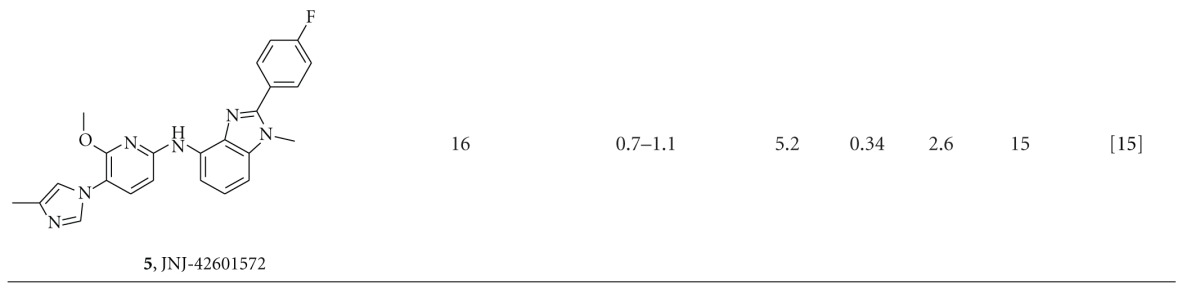

^a^LE, LLE, and LELP were calculated using the formulas given in the Methods section. *In vitro* potency values were taken from the indicated references, except for **3**, for which A*β*42 IC_50_ was determined internally.
